# Pre-clinical modeling of cutaneous melanoma

**DOI:** 10.1038/s41467-020-15546-9

**Published:** 2020-06-05

**Authors:** Vito W. Rebecca, Rajasekharan Somasundaram, Meenhard Herlyn

**Affiliations:** The Wistar Institute, Melanoma Research Center, Philadelphia, PA USA

**Keywords:** Cancer models, Cancer therapy, Melanoma

## Abstract

Metastatic melanoma is challenging to manage. Although targeted- and immune therapies have extended survival, most patients experience therapy resistance. The adaptability of melanoma cells in nutrient- and therapeutically-challenged environments distinguishes melanoma as an ideal model for investigating therapy resistance. In this review, we discuss the current available repertoire of melanoma models including two- and three-dimensional tissue cultures, organoids, genetically engineered mice and patient-derived xenograft. In particular, we highlight how each system recapitulates different features of melanoma adaptability and can be used to better understand melanoma development, progression and therapy resistance.

## Abstract (web summary)

Despite the new targeted and immunotherapies for metastatic melanoma, several patients show therapeutic plateau. Here, the authors review the current pre-clinical models of cutaneous melanoma and discuss their strengths and limitations that may help with overcoming therapeutic plateau.

## Introduction

Although melanoma constitutes ~5% of all skin cancers, it accounts for >75% of skin cancer deaths. The 5-year relative survival rate of patients with localized or regional disease is 98% and 64%, respectively. In contrast, the 5-year survival rate drops to 23% in patients with metastatic (stage IV) melanoma. One of the most common complications experienced by stage IV melanoma patients is metastasis to the brain^1,2^, which is diagnosed in >60% of cases and is identified in up to 80% of patients at autopsy^3^. Melanoma manifests from the malignant transformation of melanocytes, cells derived from neural crest stem cells (NCSCs) that produce melanin in the skin^4^. The NCSC origin of melanocytes underlies the ability of melanoma cells to both migrate to and thrive in the brain and other major organs, including lungs. There are several types of melanoma that fall into one of three categories: (1) cutaneous melanoma, (2) mucosal melanoma, or (3) ocular melanoma. Cutaneous melanoma (hereafter melanoma) is the most prevalent type and will be the focus of this review. Four major genetically defined subgroups stratify the patient population into those whose melanoma possess (1) activating *BRAF*^V600^ mutations (~50% of patients)^5^, (2) *NRAS* mutations (15–20% of patients)^6^, (3) those with inactivating mutations of *NF-1* (~10%, mutually exclusive with *BRAF*), and (4) those with wild-type *BRAF*, wild-type *NRAS* and wild-type *cKit* (30–35% of patients)^7^. Metastatic melanoma was historically viewed as an untreatable disease until the revolutionary FDA approvals in 2011 and 2014 of targeted- and immune-based therapeutic strategies with notable activity. For patients with *BRAF*^V600E/K^ mutant melanoma, significant efficacy has been observed with the combination of a BRAF inhibitor and a MEK inhibitor, with a response rate of ~76%^8^. Despite this remarkable activity, >80% of patients relapse on the BRAF/MEK inhibitor cocktail, leaving them eligible only for immunotherapy with anti-PD-1 and anti-CTLA4 blockade strategies^9^. Patients with wild-type *BRAF* (~35% of patients) do not have targeted therapy strategies that display significant clinical efficacy^10^. However, combination MEK inhibition and CDK4/6 inhibition has shown activity pre-clinically in the *BRAF* wild-type setting, and clinical testing of this approach is under way. The activity of combination checkpoint inhibitor therapy using anti-PD-1 and anti-CTLA-4 antibodies has demonstrated long-lasting responses in a subset of patients and represents a therapeutic strategy suitable for all genotypes^11^. However, 60–70% of melanoma patients do not respond to checkpoint inhibitor therapy due to toxicity, intrinsic resistance, and other reasons not completely understood, leaving surgery, radiation, chemotherapy, and clinical trials to combat the persisting melanoma cells that do not respond to current standard-of-care strategies^12^.

This is the clinical predicament in 2020 for most patients with metastatic melanoma and represents the challenge clinicians and investigators are attempting to overcome: therapeutic plateau followed by relapse and mortality. Once disseminated, there are only a few melanoma patients who experience long-lasting cures from current targeted- and immune therapies. Our evolving understanding of the genetic and non-genetic mechanisms driving melanoma dissemination, therapy resistance and mortality reveals phenotypic plasticity, inter- and intra-tumoural heterogeneity, and the microbiome among the key drivers^13^. The dynamic interactions of melanoma cells with other cellular and acellular constituents of the tumor microenvironment (TME) provide additional mechanisms of homeostatic regulation critical to therapy efficacy^14^. Recent technological advancements have only now allowed for characterization of melanoma plasticity and heterogeneity; however, the role served in therapy resistance remains poorly understood^15^. Single-cell RNA sequencing approaches have begun to dissect the multicellular ecosystems that are functional in the TME, which comprise immune and non-immune compartments each with secretory and adhesion signaling landscapes that complicate targeting of melanoma cells. A nuanced observation moving to the forefront of the field is the reality that subpopulations of melanoma adopt distinct cellular identities akin to NCSCs and stromal cells heterogeneously, within different regions of the same tumor^16^. These alternative cellular states can be adopted transiently or permanently, each with implications on sensitivity to a given therapy strategy. Recent reports have characterized therapy-resistant “jackpot” melanoma cells marked by high EGFR and NGFR expression that pre-exist before therapy and drive therapeutic relapse^17^.

Functional in vitro and in vivo preclinical models of melanoma initially demonstrated the utility of small molecule BRAF inhibitors for the treatment of *BRAF*-mutant melanoma, providing the scientific rationale for BRAF inhibitor clinical trials^18,19^. These studies leveraged “traditional” melanoma cell lines established from patients and cultured on plastic. Critical for the development of future strategies that can overcome the current clinical plateau with targeted- and immune-based therapeutic strategies, the melanoma field in itself will be a model of melanoma that better recapitulates the multifarious mechanisms that drive resistance in vivo in human patients. Here, we will discuss distinctive features of melanoma, survey the major model systems leveraged in the study of therapy resistance and comment on prospective directions that may facilitate discovery of more curative therapeutic modalities. A summary of all available melanoma models is reported in Box 1.

Box 1 Modelling melanoma in vitro and in vivo Traditional 2D cell culture and 3D organoid approaches that incorporate microenvironmental elements allow for investigations of melanoma proliferation following various environmental and therapuetic conditions. Spheroid, skin reconstruct, and endothelial *trans*-membrane models enable in vitro study of migration, invasive, and metastatic dynamics. The limitations of the above models rests on their ex vivo nature. Tail vein injection and spontaneous metastasis models are in vivo approaches that allow inspection of micro- and macrometastases in target organs (i.e., lungs, liver). Sphere, organoid, PDX, and xenograft models of melanoma allow for delineation of cellular heterogeneity and plasticity of melanoma cells that only recently can be unbiasedly characterized by scRNAseq and barcoding approaches, however, limitations lie in the absence of an immune system. Co-culture approaches incorporating stromal, endothelial, and immune cell types help overcome this limitation. GEMM, immune- humanized, and autologous adoptive T-cell models currently represent the best approaches to investigate immune dynamics in response to therapy. Limitations for the GEMM and immune-humanized models lie in the lack of human cells and an autologous immune system, respectively. The isolation and expansion of tumor infiltrating leukocytes along with tumor from the same patient tumor material for autologous models is a challenging accomplishment.

## Striking features of melanoma cells

### High genetic instability

**Fig. 1:**
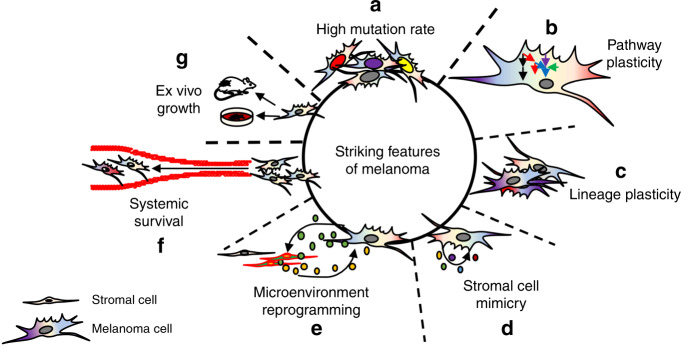
The striking features of melanoma. **a** Melanoma cells display high levels of mutational burden in cancer. **b** Melanoma cell signal transduction pathways contain significant redundancy in response to therapy, allowing for rapid signal rewiring to avoid cell death. **c** Invasive, stem-like, and proliferative cell states are distinct intra-tumoral phenotypes that drive melanoma aggressiveness. **d** Melanoma cells secrete factors akin to stromal cells, promoting melanoma cell viability in an autocrine manner. **e** Melanoma cells secrete factors that reprogram adjacent stroma which, in turn, secrete pro-tumorigenic factors that promote melanoma aggressiveness in a paracrine manner. **f** Melanoma cells can survive the harsh environment of systemic circulation, with >70% melanoma patients possessing brain metastases at autopsy. **g** Melanoma cells readily survive ex vivo, allowing for high success rate in establishing cell lines and PDX.

Melanoma incidence and manifestation are correlated with skin-type and ultraviolet radiation (UVR) exposure^20^. Allelic variation of the melanocortin 1 receptor (MC1R) is causally associated with individuals with red hair and fair skin due to a switch from eumelanin to phaeomelanin production^21,22^. Eumelanin is UV-absorbent, whereas pheomelanin is photo-unstable, which is posited to underlie the increased susceptibility to develop melanoma in individuals with MC1R variants due to the reduced UVR protection and increased reactive oxygen species activity in their skin^23^. Notably, melanomas carry the highest mutational load across human tumors as seen by cancer genome deep sequencing, at least in part, due to UVR-induced damage^24^ (Fig. 1a). Of these mutations, ~80% display canonical UV signatures (i.e., G > T or C > T transitions that are induced by UVA and UVB, respectively)^7^. UVA and UVB have been linked to serve a causal role in passenger mutations as well as ~46% of authenticated driver mutations. Likely, the large number of passenger mutations in melanoma may take potential driver roles under varying environmental or therapeutic contexts to maintain melanoma cell viability. Key mechanisms of acquired resistance to combination BRAF inhibitor and MEK inhibitor therapy include mutations in *MEK* and *NRAS*^25^; however, it is unknown whether cells expressing these mutations are pre-existing in the tumor or the mutations occur de novo. The high genetic instability of melanoma cells can be potentially exploited by targeting DNA damage repair proteins (i.e., PARP) to increase the efficacy of targeted therapy^26^.

**Fig. 2:**
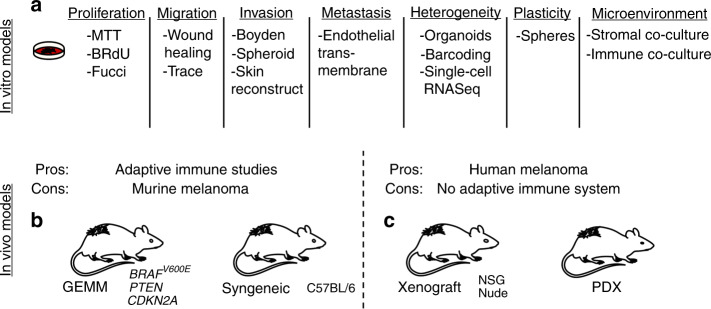
Available melanoma models. **a** A large number of in vitro models are available to investigate specific properties of melanoma cells, including proliferation, migration, invasion, metastasis, heterogeneity, plasticity, and microenvironment interactions. **b** In vivo models capable of investigating adaptive immune dynamics require murine melanoma models, whereas (**c**) models utilizing human melanoma tumor models lack functional adaptive human systems. Scientific illustration toolkits from Motifolio (www.motifolio.com) were used to generate this figure.

To better model the high genetic instability of melanoma cells in mice with intact immune systems (will be discussed further later), investigators have developed genetically engineered mouse melanoma (GEMM) cell lines that have been irradiated to account for this feature (Fig. 2).

### Pathway plasticity

Hardwired into melanoma cells is an incredibly plastic network of signal transduction pathways capable of reactivating and diverting activity from one pathway to another, allowing survival signals to be continuously transmitted in the context of targeted therapy^27^ (Fig. 1b). In the case of *BRAF*^V600^ mutant melanoma cells treated with BRAF inhibitor, the MAPK pathway is reactivated within hours in vitro and weeks in patients^28^. Although combination therapy with a BRAF inhibitor and MEK inhibitor delays MAPK pathway reactivation, it eventually occurs in the majority of cases, as well as hyperactivation of the parallel PI3K axis^29,30^. Hyperactivation of PI3K signaling in response to MAPK pathway inhibition can occur through release of negative feedback regulation of ERK phosphorylation on EGFR (i.e., at Thr^669^)^31^, as well as kinome reprogramming in response to release of feedback of ERK upon c-Myc (i.e., at Ser62)^32^. In *NRAS* mutant melanoma cells, a robust reactivation of the MAPK pathway also occurs within hours in response to MEK inhibition due to loss of negative feedback on CRAF^33^. In this context, concurrently targeting MEK and ERK, or silencing CRAF can overcome MEK inhibitor resistance. Pathway reactivation also occurs in the context of inhibitors of PI3K/AKT/mTOR, due to loss of negative feedback (i.e., through degradation of IRS-1)^34–36^. Pathway plasticity abrogates the clinical efficacy of targeted agents and warrants further investigation to identify synthetic lethality approaches that can overcome escape mechanisms. Unfortunately, current attempts to pharmacologically address pathway switching often involves therapeutic cocktails that are toxic to patients.

To model the high pathway plasticity melanoma cells display in response to targeted therapies, investigators can leverage 2D and 3D approaches to capture signaling kinetics following short-term (minutes to hours following treatment), long-term (days of treatment), and chronic (weeks to months) drug exposure times by western blotting and reverse-phase protein arrays (RPPAs). Advances in intravital imaging and multiplex in situ approaches coupled with reporters of the MAPK pathway and cell cycle also allow modeling of melanoma pathway plasticity in vivo^37,38^. Any therapuetic strategies developed against this feature of melanoma using in vitro models should be validated in patient-derived xenograft (PDX) and xenograft models before translation into humans (Fig. 2).

### Dedifferentiation

Metastatic melanoma cells display the striking ability to dedifferentiate to a variety of states under cellular stress, which drives therapy resistance and mortality^16^ (Fig. 1c). Murine implantation experiments demonstrated the intrinsically high self-renewal capacity of melanoma cells, with just one cell capable of reconstituting a heterogenous tumor in mice, a property unique among cancers whereby hundreds to thousands of cells typically need to be implanted to form a palpable tumor^39^. As melanocytes derive from NCSCs^4^, multiple laboratories have identified subsets of melanoma cells that appear to dedifferentiate and display stem-like features akin to their NCSC precursors^16^. These stem-like subpopulations display NCSC molecular features (i.e., KDM5B^16^, CD133^40–42^, CD20^43,44^, NGFR^17,45–47^, and AQP1) and biological properties (high plasticity, migratory capacity, and invasiveness) as well as a general loss of pigmentation. The ability of melanoma cells to access developmental programs bestows their remarkable adaptability to survive in a variety of hostile environments, including systemic circulation^48^, new organ sites of metastases^14^, and ex vivo in tissue culture.

To model the lineage plasticity of melanoma cells as they adopt a range of differentiated and dedifferentiated cellular states, investigators can leverage scRNAseq and single-cell RNA FISH approaches to acquire the multimarker resolution necessary to define and identify these subpopulations of melanoma cells within a given bulk tumor in vivo or a population of cells growing in a culture vessel in vitro. Investigators should determine whether vulnerabilities of these subpopulations that may present themselves through transcriptomic characterization can be validated in secondary experiments. To translate clinically, findings must optimally be mirrored in patient-derived tumor tissue and investigators should attempt to identify FDA-approved compounds that can be repurposed quickly for patient benefit.

### Stromal mimicry

Melanoma cells undergo stromal mimicry, meaning they possess the ability to secrete growth factors and cytokines normally derived from stromal fibroblasts^49,50^, monocytes^51^, macrophages, and neutrophils^52^, which promote melanoma cell viability in an autocrine loop (Fig. 1d). In addition, melanoma cells secrete a wide variety of cytokines and growth factors that also influence the tumor microenvironment in a paracrine manner^53,54^. One mechanism whereby melanoma cells achieve this commensalism-type relationship with the tumor microenvironment is through the secretion of TGF-β. Secreted TGF-β activates adjacent fibroblasts, which leads to microenvironment reprogramming associated with an increase in stromal-derived pro-tumorigenic factors (i.e., VEGF, PDGF)^55^ (Fig. 1e). Aggressive melanoma cells can also form primitive, fluid-conducting vessel-like structures when placed in three-dimensional matrices^56,57^. The in vivo biological implications of this phenotype are unclear; however, it provides another powerful example of the ability of subpopulations of melanoma to dedifferentiate to a state similar to fetal cells as survival in systemic circulation and metastatic dissemination is favored for (Fig. 1f).

Melanoma cells display a variety of immune cell properties. They possess the ability to secrete factors that negatively modulate the immune system (i.e., VEGF, IDO)^58^. Melanoma cells undergo monocyte-like phagocytosis of apoptotic cells to fuel metabolic activity^59^. Melanoma cells express major histocompatibility complex (MHC) class II molecules that allow for functional antigen presentation^60,61^. Functionally, melanoma cells operate as a primitive organ. But in contrast to a normal organ where a homeostatic balance exists between the different cell types, melanoma cells dominate the other cell types and reprogram them in favor of pro-tumorigenic functions. Critical to effectively study and develop therapeutic strategies that completely eliminate melanoma cells from a patient will be the ability of experimental models to recapitulate the spatial and environmental pressures faced by melanoma cells in the human patient.

To model stromal mimicry of melanoma, investigators can leverage co-culture 2D and 3D in vitro approaches to characterize dynamic interactions between melanoma cells and cells of the tumor microenvironment in the context of therapy and other stressful conditions (i.e., hypoxia)^62^ (Fig. 2). Window-chamber approaches can allow validation in vivo of strategies that overcome microenvironment-mediated-resistance mechanisms. Investigators should determine whether strategies that ablate the stromal features of melanoma cells can prevent metastases and the onset of resistance in a series of PDX and/or xenograft studies before translation into human patients.

### Two-dimensional (2D) melanoma cell culture models

Though in vitro 2D models are considered as “simple” relative to three-dimensional (3D) and in vivo models, all existing FDA-approved targeted-based therapies for melanoma began their journey to the clinic as classical 2D adherent cell culture models in the laboratory. In 2D models, melanoma cells are typically grown on tissue culture plates with relatively high levels of oxygen and nutrients^63^. Although exceptions exist, drugs that have little effect on the viability of melanoma cells grown in these conditions often do not have efficacy in more realistic 3D in vitro and in vivo models (as discussed later). Therefore, this approach has great utility in initial high-throughput screens to identify potential hits worth additional investigation.

The translational nature of 2D in vitro melanoma models is attributed to the high level of recapitulation that human normal melanocytes, pre-malignant nevus cells, and primary and metastatic melanoma cells reflect in culture, dependent upon the clinical stage they are derived from (Fig. 1g). Cultured melanocytes and nevus cells undergo replicative senescence, whereas melanoma cells from invasive primary and metastatic lesions grow as permanent cultures (in part due to p16 loss)^64^. Further, melanoma cells, but not melanocytes and nevus cells, form tumors in immune-deficient mice as the prime indicator of their malignant phenotype. Conventional 2D culture allows for a variety of phenotypes and striking features of melanoma to be interrogated, including cytotoxicity, proliferation (MTT, BRdU), mobility (scratch assay)^65^, invasiveness (Boyden chamber), adaptability to hypoxic microenvironments^66^, protein expression and pathway plasticity (Western blot, reverse-phase protein array) (Fig. 1b), drug sensitivity studies (high-throughput screening)^67^, drug durability (colony formation assay), molecular characterization (proteomics), and genomic/genetic characterization (RNA sequencing, whole-exome sequencing)^68^ (Fig. 2a). 2D culture also allows for the study of lineage plasticity, with key findings identifying markers of distinct melanoma lineages (Fig. 1c). Of additional benefit, monocultures are free from contaminating cells, which allows for a clear understanding of what is occurring specifically within melanoma cells relative to other cell types in the tumor microenvironment in response to a given insult, which can be accomplished through co-culture using transwell plates (Fig. 1e). A total of >2000 melanoma cell lines have been generated by the field, making this malignancy one of the most extensively studied in cancer. The extensive genetic and genomic analyses performed for most of these melanoma cell lines in systematic cancer cell-line screens as well as high-throughput drug screens have provided comprehensive information on the underlying genes and pathways responsible for driving melanoma progression and therapy escape^68^.

Although meaningful insights can be gained from 2D culture, drawbacks to this approach include lack of heterogeneity and the existence of phenotypes that are observed on plastic that do not reflect melanoma behavior in vivo, and vice versa. For example, melanoma cells proliferate much more rapidly in vitro relative to in vivo rates, likely due to high serum concentrations and stiffness of support (i.e., plastic). Genetic drift also occurs in long-term passaged cells which can lead to high variability and reduced reproducibility of the results, due to adaptations to the non-physiological conditions (i.e., oxygen and nutrient levels) in traditional 2D cell culture^66^. Short-term cultures may overcome some of these issues, but 3D models that incorporate matrices that better recapitulate in vivo microenvironment architecture may also facilitate the translatability of preclinical findings to the clinic.

## 3D melanoma cell culture models

### 3D skin reconstruct

To develop curative therapeutic strategies against melanoma, experimental models unique to melanoma are needed that faithfully recapitulate the in vivo human disease system. Of the available in vitro melanoma models that exist, the 3D skin reconstruct provides investigators with a contextually representative model^69,70^. Human skin consists of (for simplicity) an epidermis and dermis. In the epidermis are located keratinocytes and melanocytes, whereas in the dermis are located fibroblasts with a basement membrane separating the epidermis from the dermis^71^. Mouse skin differs significantly from that of human skin due to deviating cellular architecture and physiology (i.e., mouse melanocytes are located in hair follicles)^72^. This limits the use of mouse models to investigate cell–matrix and cell–cell interactions between melanoma cells and other cell types. The 3D skin reconstruct model is a unique tool for the study of melanoma behavior in human skin. The 3D skin reconstruct consists of an “epidermis” containing stratified, differentiated keratinocytes, a functional basement membrane, and a “dermis” containing fibroblasts embedded in the most prevalent extracellular matrix (ECM) found in the human skin, collagen I^70^. When melanocytes grow in the 3D skin reconstruct, they display physiological features of melanocyte homeostasis and melanoma progression observed in the skin of human patients. When incorporating melanoma cells into the 3D skin reconstruct, they exhibit characteristics analogous to the aggressiveness in the human melanoma patient. Critical to the success of this model is the ability to viably culture melanocytes, melanoma cells, keratinocytes, and fibroblasts for use in the 3D skin reconstruct. Melanocytes can be derived from human skin, but can also come from embryonic stem cells (ESCs)^73^ or induced pluripotent stem (iPS) cells^74^.

The unique architecture and composition of the 3D skin reconstruct allows for careful investigations into autocrine and paracrine loops between melanoma cells, keratinocytes, and fibroblasts, as well as physical cell–cell contact and tensile forces of ECM fibers^75^. 3D immune skin reconstruct models have also been developed, which contain melanoma cells, autologous T cells, and fibroblasts that can be allogeneic^76^. This approach allows visualization and quantitation of T-cell-mediated tumor cell killing. For preparation, human fibroblasts are suspended in collagen. Melanoma cells are then layered onto the collagen/fibroblast matrix and allowed to grow overnight (layer 2). They are then covered with a cell-free collagen layer, and then an equal number of immune T cells (antitumor reactive cytotoxic T cells) are mixed with fibroblasts in collagen and layered on top (layer 4). Four to 9 days after the addition of T cells, the reconstructs are fixed with formalin and processed for histological evaluation^70^. The assay can also be used with NK cells instead of T cells or one can use the total peripheral blood mononuclear cell population. A major advantage of 3D immune assays is the “natural” setting, in which malignant, stromal, and immune cells migrate toward each other through layers of collagen. Chemoattraction and effective T cell-mediated tumor cell killing can be assessed simultaneously. Their disadvantage is the need for autologous pairs of T cells and tumor cells, which can be readily produced for melanoma but are more challenging for epithelial tumors.

### Spheroids

In addition to the 3D skin reconstruct, there are other 3D models that capture the heterogeneity and complex intracellular interactions of a tumor similar to the in vivo conditions in human melanoma patients. Spheroids are aggregates of cells that are embedded in collagen type I, to which the outer cells adhere and invade into. From a biologically early stage, melanoma cells form spheroids but do not invade much into collagen, displaying a radial growth phase phenotype. In contrast, cells from metastatic lesions are highly invasive (vertical growth phase phenotype) and can be used to assess therapy responses to signaling inhibitors. Spheroids generally have a hypoxic core and the relative percentage of cells within the core that are dividing or undergoing apoptosis varies among different melanomas^77^. The highest viability and proliferative activity of the malignant cells are generally found at the periphery, but the collagen-invading cells in the “dendrites” of a spheroid undergo a phenotype switch to a more invasive, less proliferative state. When fibroblasts and melanoma cells are co-embedded in collagen, the fibroblasts typically infiltrate into the spheroids. Overall, spheroids reflect the general conditions in a solid tumor with matrix deposition, heterogeneous growth states, and presence of cancer stem-like cells^78^. Semi-high-throughput screens in 384-well plates can automatically evaluate viability and invasion^79,80^. Thus, spheroid culture represents a valuable cellular condition that is complementary to conventional 2D culture conditions, reflects a solid tumor in vivo, and should therefore serve as a tool to identify treatment strategies worth investigating in vivo. Limitations of the spheroid approach include being comprised cells grown in 2D culture, inability to propagate additional spheroids for longitudinal studies, and limited number of cell types that can be concurrently co-cultured.

### Organoids

Organoids represent an ex vivo 3D model capable of self-propagating that, in addition to the similar architecture of spheroids, incorporates autologous lymphoid, myeloid, and other host cell populations that are retained in human tumor isolates. The presence of the different cell lineages allows for powerful investigation into how therapy may impact the immune compartment to stimulate a greater antitumor response. A recent report leveraging this organoid platform demonstrated that TBK1/IKKε inhibition increased the efficacy of PD-1 blockade and strongly correlated with in vivo tumor response^81^. Organoids also allow a high-throughput 384-well format to test the efficacy of single-agent and combinatorial therapeutic strategies. Spheroids are more “user” friendly compatible with most established cell lines. In contrast, organoid cultures initially require careful establishment before routine use. However, the organoid approach provides the strength of using cellular material that has not been adapted to culture on plastic, and therefore will better recapitulate melanoma in human patients. The limitations of organoids lie in the fact that a relatively small number of cells are sampled for their formation, which may have implications on their level of clonality and heterogeneity they can maintain relative to the clonality and heterogeneity within the original patient tumor population. One can begin to overcome this limitation and increase the translational potential of this model by establishing organoid cultures from material derived from multiple areas of the same tumor and/or other metastatic sites to better capture the clonality and heterogeneity that existed with a given patient’s tumor.

### Capillary network formation

To recapitulate in vivo vasculature dynamics and molecular mechanisms that confer the ability to survive in systemic circulation (Fig. 1f), the capillary network formation model begins when adherent endothelial cells are first covered with collagen type-1 followed by a second layer of collagen mixed with fibroblasts. As the fibroblasts begin to constrict the collagen, they attract the endothelial cells, which penetrate the first collagen layer to migrate toward the fibroblasts^82,83^. As soon as the endothelial cells establish contact with the fibroblasts, they form round structures, which develop by day 5 into capillary networks. When melanoma cells are co-embedded with the fibroblasts into the upper collagen layer, they may stimulate or inhibit network formation, which depends on the growth factors and matrix proteins released by the malignant cells, and differs between cell lines. This assay, while requiring longer incubation times, appears more robust than the Matrigel endothelial network assay, in which endothelial cells are embedded in the matrix for just a few hours precluding any functional vessel formation^84^. The capillary network formation model allows for the investigation of pro-angiogenic mechanisms leveraged by melanoma cells to survive in environmentally stressful conditions, which may facilitate the development of therapuetic modalities that ablate microenvironment-dependent resistance mechanisms and prevent the ability of melanoma cells to enter vessels to metastasize. The limitations of this approach lie in the lack of functional vessel formation, which may hinder intra- and extravasation characterization. As with all of the above in vitro models, any potential therapuetic strategies that can ablate the ability of melanoma cells to hijack angiogenic programs to facilitate metastatic dissemination and therapy escape should be validated in a series of PDX and xenograft models in order for potential translation into human patients.

## Murine in vivo melanoma models

### Genetically engineered mouse and allograft models of melanoma

Genetically engineered mouse models (GEMMs) that enable the spontaneous formation of melanoma have allowed for key insight into melanomagenesis (Fig. 2b). Many of the early GEMMs were modeled on the knowledge of melanocyte developmental biology, environmental melanoma causative factors and frequently mutated melanoma driver genes (i.e., *BRAF, CDKN2A, CDK4, GNAQ, NRAS*)^85^. Ultraviolet (UV) radiation is the main environmental risk factor for melanoma, which underlies its substantial use in investigations of the underlying biology for melanomagenesis. Another strategy to identify pathways involved in the initiation of melanoma is via the use of the carcinogen 7,12-dimethylbenz(a)anthracene (DMBA)), which can be correlated to human etiology of melanoma^86^. To target melanocytes specifically for transformation, tissue-specific genes including *Tyr* and *MITF* were leveraged^86^. Incorporation of UV light and/or DMBA could accelerate melanoma development in different GEMM models and increase their immunogenicity for use in studies of immunotherapies^87,88^. The *CDKN2A* genes encodes two overlapping tumor suppressors (*p16INK4A* and *p14ARF*), which could be targeted by SV40 T-antigen expression using the melanocyte-specific Tyr promoter. However, loss of *CDKN2A* alone does not robustly generate cutaneous melanoma. Transgenic mice were generated by overexpressing the HGF-cMET signaling axis under the control of the murine *MT1* gene promoter, which constitutively activates the RAS/RAF/MEK/ERK signal transduction pathway. Tyr-Cre approaches targeting *NRAS* or *BRAF* genes also successfully generate cutaneous melanomas after concurrent deletion of tumor suppressor genes *Ink4a/Arf* or *Pten*. The TCGA reveals the distinct heterogeneity and high mutational burden of melanoma, which raises a limitation as GEMM models may not reflect the mutational burden found in human patient tumors. This caveat of GEMM models can be improved by including etiological factors that avoid artificial strong driver oncogenes and allow for tumor evolution (i.e., allowing for loss of heterozygosity and disease progression from early stage). An example of this lies in the ability to express and/or deactivate genes of interest in a time-dependent as well as tissue-specific manner^86^. This technology, which most commonly relies on the Cre-recombinase/LoxP system, allows for the generation of elegant mouse models that conditionally knockout and/or overexpress multiple genes in a controllable fashion. Using this approach, the McMahon and Bosenburg laboratories generated the constitutively activated BrafV600E mutant with simultaneous deletion of PTEN mouse model, which is conditionally expressed in a 4-OHT-dependent and melanocyte-specific manner^89^. The use of GEMMs and allograft approaches to study melanoma suffers from the drawbacks mentioned, but it does allow the study of immune cell and tumor cell interactions/dynamics in immunocompetent mice.

### Non-murine in vivo melanoma models

In addition, melanoma models in other species including canine and zebrafish also allow for unique opportunities for investigating in vivo immune consequences of therapy and high-throughput in vivo approaches, respectively. Immense progress has been recently made with the use of zebrafish for melanoma research from the laboratories of Leonard Zon, Richard White, and Elizabeth Patton^90,91^. A few discoveries from this aquatic model system include the interaction of p53 and BRAF to produce melanoma, and the elucidation of developmental pathways in neural crest cells that have implications on melanoma formation.

### Xenograft melanoma models

In vivo models of melanoma allow for physiologic components not fully present in existing in vitro models. Traditionally, in vivo melanoma models are comprised xenografting tumor cell lines, initially established and cultured on plastic, into immunocompromised mice. Once engrafted, human melanoma cells develop dynamic physical and secretory interactions with murine stroma, lymphatic, and blood vasculature allowing for the study of melanoma dynamics in vivo. Traditionally, preclinical testing of drug efficacy in xenograft models relies on the impact on tumor growth and/or metastasis to visceral organs (i.e., lungs, liver). Some melanoma cell lines have a high metastatic potential and can form spontaneous metastases in the lungs following subcutaneous injection. Serial passaging in mice of cell lines capable of metastasizing to the lungs can eventually select for aggressive subpopulations of melanoma cells with a higher capacity to form spontaneous metastases in the lung, as is the case for the WM1205Lu and WM451Lu cell lines relative to the parental WM793B and WM164, respectively^63^.

If a cell line of interest has low metastatic potential, cells can be injected into the tail vein or intracardiac to “force” metastasis to the lungs and brain, respectively. Although metastases at these visceral sites will form, the cells present will not have gone through critical steps in the classical metastatic cascade (i.e., intravasation, survival in circulation, extravasation) and therefore not recapitulate true metastases in human patients. An additional limitation of the xenograft approach are the artifacts that arise from passaging melanoma cell lines ex vivo selects for subpopulations that adapt to non-physiological 2D culture condition and do not necessarily reflect those most active in vivo^66^. The consequence of this selection is the poor reproducibility observed between the results in the laboratory and therapy outcome in the clinic^92^. To address this shortcoming, a technique has been developed whereby tumor pieces are biopsied from patients and xenografted directly into immune-deficient mice, never touching plastic to retain as much of the in vivo characteristics present when still in the human patient. Patient-derived xenograft (PDX) models have been demonstrated to be superior for tumor biology studies due to the level of heterogeneity maintained in the in vivo setting^93^ (Fig. 2b). The heterogeneity of PDX models, in part, may also depend on the number of tumor sources, which can be similar to cell-line based xenograft models. A recent study compared cell-line-derived xenografts (CDX) to PDX and found significant differences in hypoxia-regulated gene expression, likely attributed to the 2D cell culture adaptions to non-physiologic levels of oxygen and alter the fitness of cells when reimplanted in vivo^66^. Limitations to xenograft and PDX models lie in the necessity to utilize mice that are immunocompromised or immunodeficient. This poses challenges to the investigation of resistance mechanisms to immune checkpoint inhibitors as well as the potential role of the immune system in efficacy of targeted therapy.

To address these shortcomings for preclinical research into antitumor immune responses, laboratories in the melanoma field including our own have developed approaches to utilize adoptive T-cell therapy with PDX^94^, as well as immune-humanized mouse tumor models. Studies leveraging autologous immune-humanized mice incorporate adoptive cell transfer of tumor-infiltrating T cells and tumor cells from the same patient^95^, which have so far identified continuous presence of interleukin-2 (IL-2) in the antitumor activity of T cells^96^. Advantages of such models are the ability to experimentally interrogate in vivo interactions of human tumor cells with human immune cells in immunodeficient mice. Briefly, functional human immune systems are reconstituted in immunodeficient mice with the use of human CD34+ cord blood cells, or engraftment of human fetal thymus tissue under the renal capsule and tail-vein injection of fetal liver-derived CD34+ cells^97,98^. The quality, yield, and limited potential of CD34 + cells to reconstitute physiologically relevant levels of distinct human immune cell compartments are a major issue when using human cord blood-derived cells, which can be overcome with the use of fetal liver-derived CD34 + cells. In both instances (cord blood- or fetal liver-derived CD34 + cells), the major human leukocyte antigen (HLA) allele of the CD34 + cell donor must match the major HLA allele of the tumor cells. Our laboratory has successfully established over 50 batches of immune-humanized mice with either human cord blood-derived CD34 + cells or fetal tissue-derived CD34 + cells. Renal grafting of fetal tissue from the same donor of CD34 + cells minimizes alloreactivity and graft-versus-host (GVH)-related issues. The immune-humanization with fetal tissue at the moment allows for superior recapitulation of immune cell and tumor cell interactions in vivo, however the necessity of fetal tissue makes this model difficult to sustain, impossible in certain countries, and unfortunately does not provide autologous immune-humanized mice with respect to a given tumor. To overcome this hurdle, our laboratory is developing an induced pluripotent stem cell (iPS) model to generate CD34 + stem cells to humanize mice. The advantage of the iPS approach is the donor CD34 + cells are fully autologous with the tumor cells as they are both derived from the same individual. Although not yet fully optimized, we believe this approach will provide the field with a valuable model to finally begin assessing immune dynamics in response to therapy using PDX models that reflect the heterogeneity and clonal diversity of human melanomas in vivo.

### Conclusions and future directions

The overall goal of the melanoma field is to develop efficacious therapeutic strategies to cure every melanoma patient. To achieve this, researchers will need improved melanoma models that optimally mimic human melanoma development, heterogeneity, plasticity, progression, and also possess molecular characteristics unique to each patient. Given the breadth of models available, researchers have a large selection of ever more sophisticated approaches that facilitate the logical exploration of underlying mechanisms of melanoma metastasis and therapy escape. For example, if a novel gene is identified as governing melanoma migration in 2D culture models, researchers can further characterize the robustness of this finding in 3D spheroid and organoid cultures to determine whether validation in more complex and costly mouse models is warranted (Fig. 2). Ultimately, models are not “real”, but rather are simplified representations of specific biological and molecular phenomena found in melanoma cells within human patients. To best understand a given biological or molecular process, multiple models should be leveraged to differentiate robust findings that may translate to the human condition from encouraging data that may be due to artifact.

**Fig. 3:**
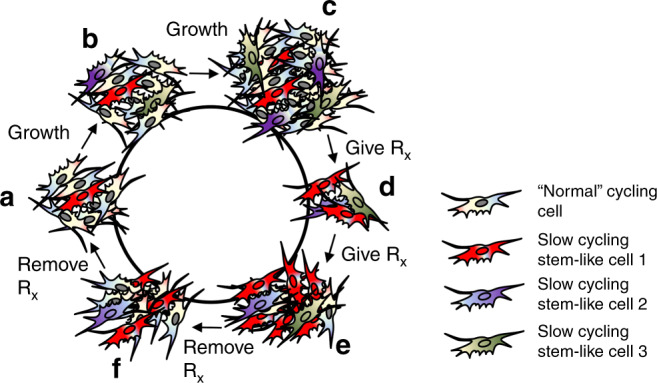
Minimal residual disease epitomizes the clinical challenge of heterogeneity and tumor plasticity. **a** A small subpopulation of melanoma cells possess stem-like molecular and biological properties and undergo cellular proliferation at a slower rate than the rest (**b**, **c**) of the population. **d** Upon the addition of therapy (Rx), the bulk of the tumor is eliminated, except the stem-like subpopulation (minimal residual disease). **e** Under continuous therapy, the stem-like cells continue to proliferation and have the capacity to birth non-stem-like progeny. **f** Upon termination of therapy, the stem-like cells will again become scarce as the “normal” cycling cells continue to proliferate at a higher extent.

Gaining wider attention is the existence of subpopulations of melanoma that are never eliminated by therapy, despite imaging and pathological analyses concluding complete response. This minimal residual disease drives therapy relapse in patients and represents one area in need of the best melanoma models dove tailed with the most sophisticated molecular and genetic techniques to unlock vulnerabilities in these distinct subpopulations of melanoma that will allow for their specific elimination (Fig. 3a–f). Perhaps the use of PDX models of melanoma in autologous immune-humanized mice will be the ideal approach to interrogate immune- and non-immune-dependent mechanisms that allow residual disease to persist through therapy and eventually drive therapy relapse. Nonetheless, in vitro assays coupled with scRNAseq and barcoding approaches will continue to provide critical insights into the development of therapy resistance that will guide how the field addresses therapy resistance. Improved models are required capable of maintaining the in vivo physiologic pressures that allow melanoma cells to exhibit identical behavior as found in human patients. The complex and dynamic interactions between melanoma cells and other cell types in the TME are currently only studied in vitro through co-culture of 1–2 additional cell types at a time. Advances in bioprinting and generation of artificial organs may allow the development of in vitro models that possess all of the relevant cell types and 3D architecture to study melanoma biology and therapeutic resistance. A hurdle to the use of GEMM, xenograft, and PDX mouse models is the cost of largescale use. A potential replacement to these in vivo models may lie in the high-throughput use of organoids for screening that can be validated in vivo for robust hits. The development of noninvasive approaches, including liquid biopsies, and the investigation of circulating tumor cells to interrogate treatment efficacy in patients can also empower physicians with the ability to modify treatment regimens in real time to improve patient overall survival.

## References

[1] Schmidberger H (2018). Long-term survival of patients after ipilimumab and hypofractionated brain radiotherapy for brain metastases of malignant melanoma: sequence matters. Strahlentherapie und Onkol..

[2] Arheden, A. et al. Real-world data on PD-1 inhibitor therapy in metastatic melanoma. *Acta Oncologica* **58**, 962–988 (2019). 10.1080/0284186X.2019.1620966 31151362

[3] Fischer GM (2019). Molecular profiling reveals unique immune and metabolic features of melanoma brain metastases. Cancer Discov..

[4] Cramer SF (1991). The origin of epidermal melanocytes. Implications for the histogenesis of nevi and melanomas. Arch. Pathol. Lab. Med..

[5] Chang DZ (2004). Clinical significance of BRAF mutations in metastatic melanoma. J. Transl. Med..

[6] Eskandarpour M (2003). Frequency of UV-inducible NRAS mutations in melanomas of patients with germline CDKN2A mutations. J. Natl Cancer Inst..

[7] Hodis E (2012). A landscape of driver mutations in melanoma. Cell.

[8] Dummer R (2018). Overall survival in patients with BRAF-mutant melanoma receiving encorafenib plus binimetinib versus vemurafenib or encorafenib (COLUMBUS): a multicentre, open-label, randomised, phase 3 trial. Lancet Oncol..

[9] Schreuer M (2017). Combination of dabrafenib plus trametinib for BRAF and MEK inhibitor pretreated patients with advanced BRAF(V600)-mutant melanoma: an open-label, single arm, dual-centre, phase 2 clinical trial. Lancet Oncol..

[10] Johnpulle RA, Johnson DB, Sosman JA (2016). Molecular targeted therapy approaches for BRAF wild-type melanoma. Curr. Oncol. Rep..

[11] Rogiers A (2019). Long-term survival, quality of life, and psychosocial outcomes in advanced melanoma patients treated with immune checkpoint inhibitors. J. Oncol..

[12] Jerby-Arnon L (2018). A cancer cell program promotes T cell exclusion and resistance to checkpoint blockade. Cell.

[13] Elkrief A (2019). Antibiotics are associated with decreased progression-free survival of advanced melanoma patients treated with immune checkpoint inhibitors. Oncoimmunology.

[14] Kaur A (2016). sFRP2 in the aged microenvironment drives melanoma metastasis and therapy resistance. Nature.

[15] Bai, X., Fisher, D. E. & Flaherty K. T. Cell-state dynamics and therapeutic resistance in melanoma from the perspective of MITF and IFNgamma pathways. *Nat. Rev. Clin. Oncol.* **16**, 549–562 (2019). 10.1038/s41571-019-0204-6 PMC7185899 30967646

[16] Roesch A (2010). A temporarily distinct subpopulation of slow-cycling melanoma cells is required for continuous tumor growth. Cell.

[17] Shaffer SM (2017). Rare cell variability and drug-induced reprogramming as a mode of cancer drug resistance. Nature.

[18] Sharma A (2005). Mutant V599EB-Raf regulates growth and vascular development of malignant melanoma tumors. Cancer Res..

[19] Liu J (2007). Oncogenic BRAF regulates beta-Trcp expression and NF-kappaB activity in human melanoma cells. Oncogene.

[20] Chang C (2014). More skin, more sun, more tan, more melanoma. Am. J. Public Health.

[21] Chen S (2017). Palmitoylation-dependent activation of MC1R prevents melanomagenesis. Nature.

[22] Valverde P (1996). The Asp84Glu variant of the melanocortin 1 receptor (MC1R) is associated with melanoma. Hum. Mol. Genet..

[23] Nasti TH, Timares L (2015). MC1R, eumelanin and pheomelanin: their role in determining the susceptibility to skin cancer. Photochemistry Photobiol..

[24] Buttner R (2019). Implementing TMB measurement in clinical practice: considerations on assay requirements. ESMO Open..

[25] Nazarian R (2010). Melanomas acquire resistance to B-RAF(V600E) inhibition by RTK or N-RAS upregulation. Nature.

[26] Possik PA (2014). Parallel in vivo and in vitro melanoma RNAi dropout screens reveal synthetic lethality between hypoxia and DNA damage response inhibition. Cell Rep..

[27] Smith LK, Rao AD, McArthur GA (2016). Targeting metabolic reprogramming as a potential therapeutic strategy in melanoma. Pharmacol. Res..

[28] Villanueva J (2010). Acquired resistance to BRAF inhibitors mediated by a RAF kinase switch in melanoma can be overcome by cotargeting MEK and IGF-1R/PI3K. Cancer Cell..

[29] Johannessen CM (2010). COT drives resistance to RAF inhibition through MAP kinase pathway reactivation. Nature.

[30] Moriceau G (2015). Tunable-combinatorial mechanisms of acquired resistance limit the efficacy of BRAF/MEK cotargeting but result in melanoma drug addiction. Cancer Cell..

[31] Mirzoeva OK (2009). Basal subtype and MAPK/ERK kinase (MEK)-phosphoinositide 3-kinase feedback signaling determine susceptibility of breast cancer cells to MEK inhibition. Cancer Res..

[32] Duncan JS (2012). Dynamic reprogramming of the kinome in response to targeted MEK inhibition in triple-negative breast cancer. Cell.

[33] Rebecca VW (2014). Vertical inhibition of the MAPK pathway enhances therapeutic responses in NRAS-mutant melanoma. Pigment Cell Melanoma Res..

[34] Zitzmann K (2010). Compensatory activation of Akt in response to mTOR and Raf inhibitors—a rationale for dual-targeted therapy approaches in neuroendocrine tumor disease. Cancer Lett..

[35] Wan X, Harkavy B, Shen N, Grohar P, Helman LJ (2007). Rapamycin induces feedback activation of Akt signaling through an IGF-1R-dependent mechanism. Oncogene.

[36] Sun SY (2005). Activation of Akt and eIF4E survival pathways by rapamycin-mediated mammalian target of rapamycin inhibition. Cancer Res..

[37] Hirata E (2015). Intravital imaging reveals how BRAF inhibition generates drug-tolerant microenvironments with high integrin beta1/FAK signaling. Cancer Cell..

[38] Teh JLF (2018). In vivo E2F reporting reveals efficacious schedules of MEK1/2-CDK4/6 targeting and mTOR-S6 resistance mechanisms. Cancer Discov..

[39] Quintana E (2008). Efficient tumour formation by single human melanoma cells. Nature.

[40] Shidal, C., Singh, N. P., Nagarkatti, P. & Nagarkatti, M. MicroRNA-92 expression in CD133 + melanoma stem cells regulates immunosuppression in the tumor microenvironment via integrin-dependent activation of TGF-beta. *Cancer Res*. **79**, 3622–3635 (2019). 10.1158/0008-5472.CAN-18-2659 PMC6635067 31015227

[41] Frank NY (2005). ABCB5-mediated doxorubicin transport and chemoresistance in human malignant melanoma. Cancer Res..

[42] Klein WM (2007). Increased expression of stem cell markers in malignant melanoma. Mod. Pathol..

[43] Fang D (2005). A tumorigenic subpopulation with stem cell properties in melanomas. Cancer Res..

[44] Zabierowski SE, Herlyn M (2008). Melanoma stem cells: the dark seed of melanoma. J. Clin. Oncol..

[45] Redmer T (2014). The nerve growth factor receptor CD271 is crucial to maintain tumorigenicity and stem-like properties of melanoma cells. PLoS ONE.

[46] Fallahi-Sichani M (2017). Adaptive resistance of melanoma cells to RAF inhibition via reversible induction of a slowly dividing de-differentiated state. Mol. Syst. Biol..

[47] Mehta A (2018). Immunotherapy resistance by inflammation-induced dedifferentiation. Cancer Discov..

[48] Boisvert-Adamo K, Longmate W, Abel EV, Aplin AE (2009). Mcl-1 is required for melanoma cell resistance to anoikis. Mol. Cancer Res..

[49] Loffek S (2005). High invasive melanoma cells induce matrix metalloproteinase-1 synthesis in fibroblasts by interleukin-1alpha and basic fibroblast growth factor-mediated mechanisms. J. Investig. Dermatol..

[50] Brenner M, Degitz K, Besch R, Berking C (2005). Differential expression of melanoma-associated growth factors in keratinocytes and fibroblasts by ultraviolet A and ultraviolet B radiation. Br. J. Dermatol..

[51] Graeven U, Herlyn M (1992). In vitro growth patterns of normal human melanocytes and melanocytes from different stages of melanoma progression. J. Immunother..

[52] Peng HH, Liang S, Henderson AJ, Dong C (2007). Regulation of interleukin-8 expression in melanoma-stimulated neutrophil inflammatory response. Exp. Cell Res..

[53] Whipple CA, Brinckerhoff CE (2014). BRAF(V600E) melanoma cells secrete factors that activate stromal fibroblasts and enhance tumourigenicity. Br. J. Cancer.

[54] Busse A, Keilholz U (2011). Role of TGF-beta in melanoma. Curr. Pharm. Biotechnol..

[55] Lazar-Molnar E, Hegyesi H, Toth S, Falus A (2000). Autocrine and paracrine regulation by cytokines and growth factors in melanoma. Cytokine.

[56] Rodewald, A. K. et al. Eight autopsy cases of melanoma brain metastases showing angiotropism and pericytic mimicry. Implications for extravascular migratory metastasis. *J. Cutaneous Pathol*. **46**, 570–578 (2019). 10.1111/cup.13465 30927294

[57] Hendrix MJ (2016). Tumor cell vascular mimicry: novel targeting opportunity in melanoma. Pharmacol. Therapeut..

[58] Lacal PM (2000). Human melanoma cells secrete and respond to placenta growth factor and vascular endothelial growth factor. J. Investig. Dermatol..

[59] Lugini L (2003). Potent phagocytic activity discriminates metastatic and primary human malignant melanomas: a key role of ezrin. Lab. Investig..

[60] Rodig, S. J. et al. MHC proteins confer differential sensitivity to CTLA-4 and PD-1 blockade in untreated metastatic melanoma. *Sci. Transl. Med*. **10**, eaar3342 (2018). 10.1126/scitranslmed.aar3342 30021886

[61] Brady MS, Eckels DD, Ree SY, Schultheiss KE, Lee JS (1996). MHC class II-mediated antigen presentation by melanoma cells. J. Immunother. Emphas. Tumor Immunol..

[62] Somasundaram R (2017). Tumor-associated B-cells induce tumor heterogeneity and therapy resistance. Nat. Commun..

[63] Beaumont, K. A., Mohana-Kumaran, N. & Haass, N. K. *Modeling melanoma in vitro and in vivo*. in *Healthcare*, Vol. 2. 27–46 (Multidisciplinary Digital Publishing Institute, Basel, 2013). 10.3390/healthcare2010027 PMC4934492 27429258

[64] Sviderskaya EV (2002). p16(Ink4a) in melanocyte senescence and differentiation. J. Natl Cancer Inst..

[65] Satyamoorthy K, Li G, Vaidya B, Kalabis J, Herlyn M (2002). Insulin-like growth factor-I-induced migration of melanoma cells is mediated by interleukin-8 induction. Cell Growth Differ.: Mol. Biol. J. Am. Assoc. Cancer Res..

[66] Bhadury J (2016). Hypoxia-regulated gene expression explains differences between melanoma cell line-derived xenografts and patient-derived xenografts. Oncotarget.

[67] Park ES (2010). Integrative analysis of proteomic signatures, mutations, and drug responsiveness in the NCI 60 cancer cell line set. Mol. Cancer Therapeut..

[68] Konieczkowski DJ (2014). A melanoma cell state distinction influences sensitivity to MAPK pathway inhibitors. Cancer Discov..

[69] Ghosh S (2005). Three-dimensional culture of melanoma cells profoundly affects gene expression profile: a high density oligonucleotide array study. J. Cell. Physiol..

[70] Li, L., Fukunaga-Kalabis, M. & Herlyn, M. The three-dimensional human skin reconstruct model: a tool to study normal skin and melanoma progression. *J. Vis. Exp.* **54**, e2937 (2011). 10.3791/2937 PMC3155964 21847077

[71] Mancianti ML (1988). Growth and phenotypic characteristics of human nevus cells in culture. J. Investig. Dermatol..

[72] Silver AF, Fleischmann RD, Chase HB (1977). The fine structure of the melanocytes of the adult mouse hair follicle during their amelanotic phase (telogen and early anagen). Am. J. Anat..

[73] Gola M, Czajkowski R, Bajek A, Dura A, Drewa T (2012). Melanocyte stem cells: biology and current aspects. Med. Sci. Monit.: Int. Med. J. Exp. Clin. Res..

[74] Hosaka, C. et al. Induced pluripotent stem cell-derived melanocyte precursor cells undergoing differentiation into melanocytes. *Pigment Cell Melanoma Res*. **32**, 623–-633 (2019). 10.1111/pcmr.12779 30843370

[75] Murekatete B (2018). Targeting insulin-like growth factor-i and extracellular matrix interactions in melanoma progression. Sci. Rep..

[76] Kaur A (2019). Remodeling of the collagen matrix in aging skin promotes melanoma metastasis and affects immune cell motility. Cancer Discov..

[77] Raza A (2017). Oxygen mapping of melanoma spheroids using small molecule platinum probe and phosphorescence lifetime imaging microscopy. Sci. Rep..

[78] Tsai J (2008). Discovery of a selective inhibitor of oncogenic B-Raf kinase with potent antimelanoma activity. Proc. Natl Acad. Sci. USA.

[79] Friedrich J, Seidel C, Ebner R, Kunz-Schughart LA (2009). Spheroid-based drug screen: considerations and practical approach. Nat. Protoc..

[80] Tung YC (2011). High-throughput 3D spheroid culture and drug testing using a 384 hanging drop array. Analyst.

[81] Jenkins RW (2018). Ex vivo profiling of PD-1 blockade using organotypic tumor spheroids. Cancer Discov..

[82] Noma K (2008). The essential role of fibroblasts in esophageal squamous cell carcinoma-induced angiogenesis. Gastroenterology.

[83] Velazquez OC, Snyder R, Liu ZJ, Fairman RM, Herlyn M (2002). Fibroblast-dependent differentiation of human microvascular endothelial cells into capillary-like 3-dimensional networks. FASEB J..

[84] Khoo CP, Micklem K, Watt SM (2011). A comparison of methods for quantifying angiogenesis in the Matrigel assay in vitro. Tissue Eng. Part C., Methods.

[85] Neagu M (2016). Chemically induced skin carcinogenesis: updates in experimental models (Review). Oncol. Rep..

[86] Perez-Guijarro E, Day CP, Merlino G, Zaidi MR (2017). Genetically engineered mouse models of melanoma. Cancer.

[87] Wang J (2017). UV-induced somatic mutations elicit a functional T cell response in the YUMMER1.7 mouse melanoma model. Pigment Cell Melanoma Res..

[88] Viros A (2014). Ultraviolet radiation accelerates BRAF-driven melanomagenesis by targeting TP53. Nature.

[89] Dankort D (2009). Braf(V600E) cooperates with Pten loss to induce metastatic melanoma. Nat. Genet..

[90] White RM (2008). Transparent adult zebrafish as a tool for in vivo transplantation analysis. Cell Stem Cell.

[91] Patton EE (2005). BRAF mutations are sufficient to promote nevi formation and cooperate with p53 in the genesis of melanoma. Curr. Biol..

[92] Tveit KM, Pihl A (1981). Do cell lines in vitro reflect the properties of the tumours of origin? A study of lines derived from human melanoma xenografts. Br. J. Cancer.

[93] Harris AL, Joseph RW, Copland JA (2016). Patient-derived tumor xenograft models for melanoma drug discovery. Expert Opin. Drug Discov..

[94] Atay C (2019). BRAF targeting sensitizes resistant melanoma to cytotoxic T cells. Clin. Cancer Res..

[95] Einarsdottir BO (2018). A patient-derived xenograft pre-clinical trial reveals treatment responses and a resistance mechanism to karonudib in metastatic melanoma. Cell Death Dis..

[96] Jespersen H (2017). Clinical responses to adoptive T-cell transfer can be modeled in an autologous immune-humanized mouse model. Nat. Commun..

[97] Hu Z, Xia J, Fan W, Wargo J, Yang YG (2016). Human melanoma immunotherapy using tumor antigen-specific T cells generated in humanized mice. Oncotarget.

[98] Tonomura N, Habiro K, Shimizu A, Sykes M, Yang YG (2008). Antigen-specific human T-cell responses and T cell-dependent production of human antibodies in a humanized mouse model. Blood.

